# Dual disseminated infection with *Nocardia farcinica* and *Mucor* in a patient with systemic lupus erythematosus: a case report

**DOI:** 10.1186/1752-1947-8-376

**Published:** 2014-11-20

**Authors:** Frederik de Clerck, Florence Van Ryckeghem, Pieter Depuydt, Dominque Benoit, Patrick Druwé, Arnika Hugel, Geert Claeys, Piet Cools, Johan Decruyenaere

**Affiliations:** 1 Department of Gastroenterology and Hepatology, University Hospital, De Pintelaan 185, 9000 Ghent, Belgium; 2 Department of Medical Oncology, University Hospital, De Pintelaan 185, 9000 Ghent, Belgium; 3 Department of Intensive Care, University Hospital, De Pintelaan 185, 9000 Ghent, Belgium; 4 Department of Pathology, University Hospital, De Pintelaan 185, 9000 Ghent, Belgium; 5 Department of Microbiology, Immunology and Clinical Chemistry, Ghent University, De Pintelaan 185, 9000 Ghent, Belgium

**Keywords:** Infection, *Mucor*, Mucormycosis, *Nocardia*, Systemic lupus erythematosus

## Abstract

**Introduction:**

Infections remain a major cause of morbidity and mortality in immunocompromised patients and require early diagnosis and treatment. However, correct diagnosis and treatment are often delayed by a multitude of factors. We report what we believe to be the first case of a combined disseminated infection with *Nocardia* and *Mucor* in a patient with systemic lupus erythematosus.

**Case presentation:**

A 74-year-old Caucasian woman with systemic lupus erythematosus presented with recurrent pneumonia. Despite empirical treatment with antibiotics, her condition gradually deteriorated. Microbiological sampling by thoracoscopy revealed the presence of *Nocardia*. Despite the institution of therapy for disseminated nocardiosis, she died of multi-organ failure. A post-mortem investigation confirmed nocardiosis, but showed concomitant disseminated mucormycosis infection as well.

**Conclusion:**

Members of the bacterial genus *Nocardia* and the fungal genus *Mucor* are ubiquitous in the environment, have the ability to spread to virtually any organ, and are remarkably resistant to appropriate therapy. Both pathogens can mimic other pathologies both on clinical and radiological investigations. Invasive sampling procedures are often needed to prove their presence. Establishing a timely, correct diagnosis and a specific treatment is essential for patient survival.

## Introduction

Infections remain a major cause of morbidity and mortality in patients with systemic lupus erythematosus (SLE). These patients are at increased risk for bacterial and opportunistic infections due to intrinsic and extrinsic immunosuppression [1].

*Nocardia* species and Mucorales are ubiquitous in nature and can be found in soil, water and decaying matter [2-4]. The genus *Nocardia* belongs to a group of Gram-positive, aerobic bacteria characterized by branching, filamentous cells [2].

Mucormycosis is a fungal infection caused by members of the Mucorales, belonging to the phylum Zygomycota [3]. These organisms consist of broad and thick-walled hyphae with right-angled branching [4]. Mucorales have a characteristic tendency to invade blood vessels, causing tissue infarction, hemorrhage and necrosis [3,4]. *Nocardia* and Mucorales enter the body by direct inoculation of the skin after trauma or by inhalation [1,3,5]. Up to one third of *Nocardia* infections occur in the immunocompetent host [1,2], in contrast to Mucorales infections, which rarely occur in this population [4].

## Case presentation

A 74-year-old Caucasian woman presented to our emergency department in May 2013 with severe exertional dyspnea. She was known to have SLE and Sjögren’s syndrome, diagnosed in 1985. Her treatment consisted of hydroxychloroquine, which was then switched to low-dose oral prednisolone because of retinopathy. She was in a good overall medical condition until seven months before admission. From then on, she was hospitalized several times because of presumed respiratory infection, which temporarily responded to repeated courses of empirical antibiotic treatment.

**Figure 1:**
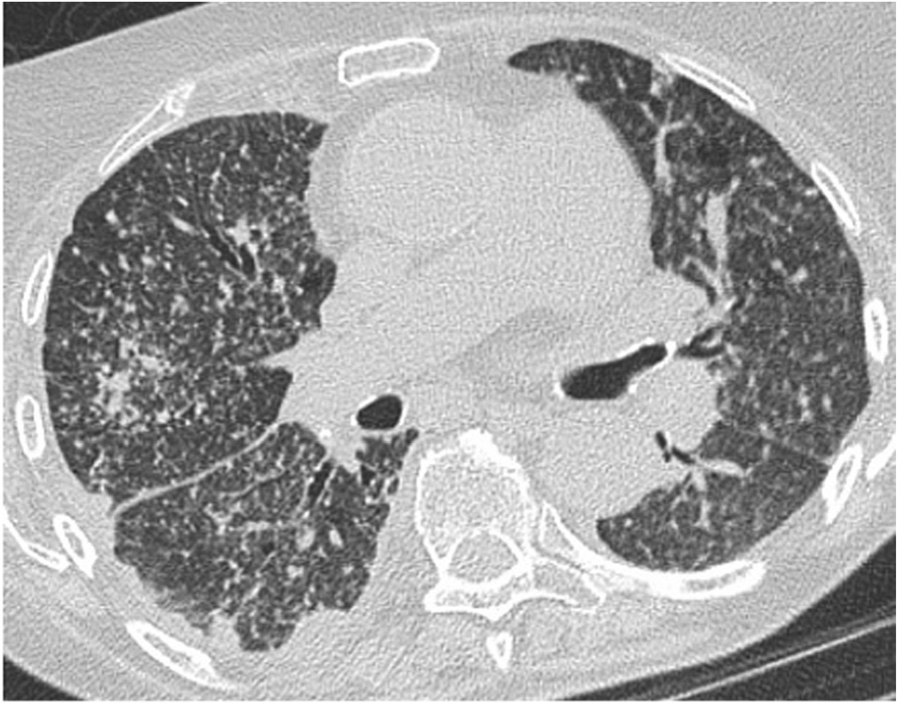
High-resolution computed tomography showing mildly enlarged lymph nodes, mostly right-sided pleural lesions, thickening of the fissures and interlobular septa, and bilateral diffuse micronodular opacities (miliary pattern).

Three months before admission, a computed tomography (CT) scan of her chest showed a mild pericardial and right-sided pleural effusion, ruling out pulmonary embolism and lung parenchymal abnormalities. Echocardiography showed concentric biventricular hypertrophic cardiomyopathy. A month later, she presented with atrial fibrillation and chest pain with elevated cardiac enzymes. We made a diagnosis of SLE perimyocarditis and started treatment with 32mg of methylprednisolone once a day combined with 100 mg of azathioprine once a day. A few weeks later, she was hospitalized because of another pneumonia of her right lower lobe. She was empirically treated with moxifloxacin. After an initial improvement she was discharged, but after a short period she presented again at our emergency department with severe exertional dyspnea, bilateral pleuritic pain, headache, night sweats, fatigue and weight loss.On admission, her vital signs were normal. Lung auscultation revealed bilateral basal crepitations and there was mild bimalleolar edema. The most prominent laboratory findings were a moderate normocytic anemia (hemoglobin 9.7g/dL) and elevated inflammatory parameters (C-reactive protein 147mg/L; white blood cell count 19760/μL). Chest radiography showed cardiomegaly, elevation of her right diaphragm, a right-sided pleural effusion and small bilateral consolidations. Echocardiography showed no pericardial effusion. Because of a highly elevated N-terminal pro-brain natriuretic peptide level (>25,000ng/L), a diagnosis of global heart failure was made and therapy was initiated. Because of her long-standing general symptoms, blind blood cultures were taken and high-resolution CT (HRCT) was planned. Two days after admission she developed fever. At that time, HRCT showed multiple mildly enlarged lymph nodes. There were multiple pleural lesions (mostly right-sided), partial compression atelectasis of her right lobe, a mild right-sided pleural effusion, thickening of the fissures and interlobular septa, and bilateral diffuse micronodular opacities (miliary pattern) (Figure 1).

Urgent bronchoscopy with bronchoalveolar lavage (BAL) led to further respiratory distress for our patient, necessitating intensive care unit (ICU) admission. Piperacillin-tazobactam was empirically started and the BAL results showed *Pseudomonas aeruginosa*, susceptible to the initiated antibiotics. Acid-fast stains were repeatedly negative (BAL and sputum). Four days later, one blood culture turned positive for *Nocardia* species. Her antibiotics were switched to trimethoprim-sulfamethoxazole combined with piperacillin-tazobactam. Intubation and mechanical ventilation proved necessary because of further respiratory deterioration. Given the findings on CT of her thorax, a diagnostic thoracoscopic lung biopsy was performed, which showed multiple sub-pleural abscesses. Overnight, an overwhelming septic shock developed with diffuse intravascular coagulation, lactic acidosis, liver failure, acute renal failure with anuria and need for high-dose noradrenaline. Continuous hemodialysis was started and amikacin was added to the antibiotics regimen. A CT scan of her brain, performed because of anisocoria, showed lesions compatible with abscess formation in her occipitotemporal area and brainstem. A tracheal aspirate and pleural biopsy confirmed the presence of massive amounts of *Nocardia* without evidence of other co-existing pathology.

**Figure 2:**
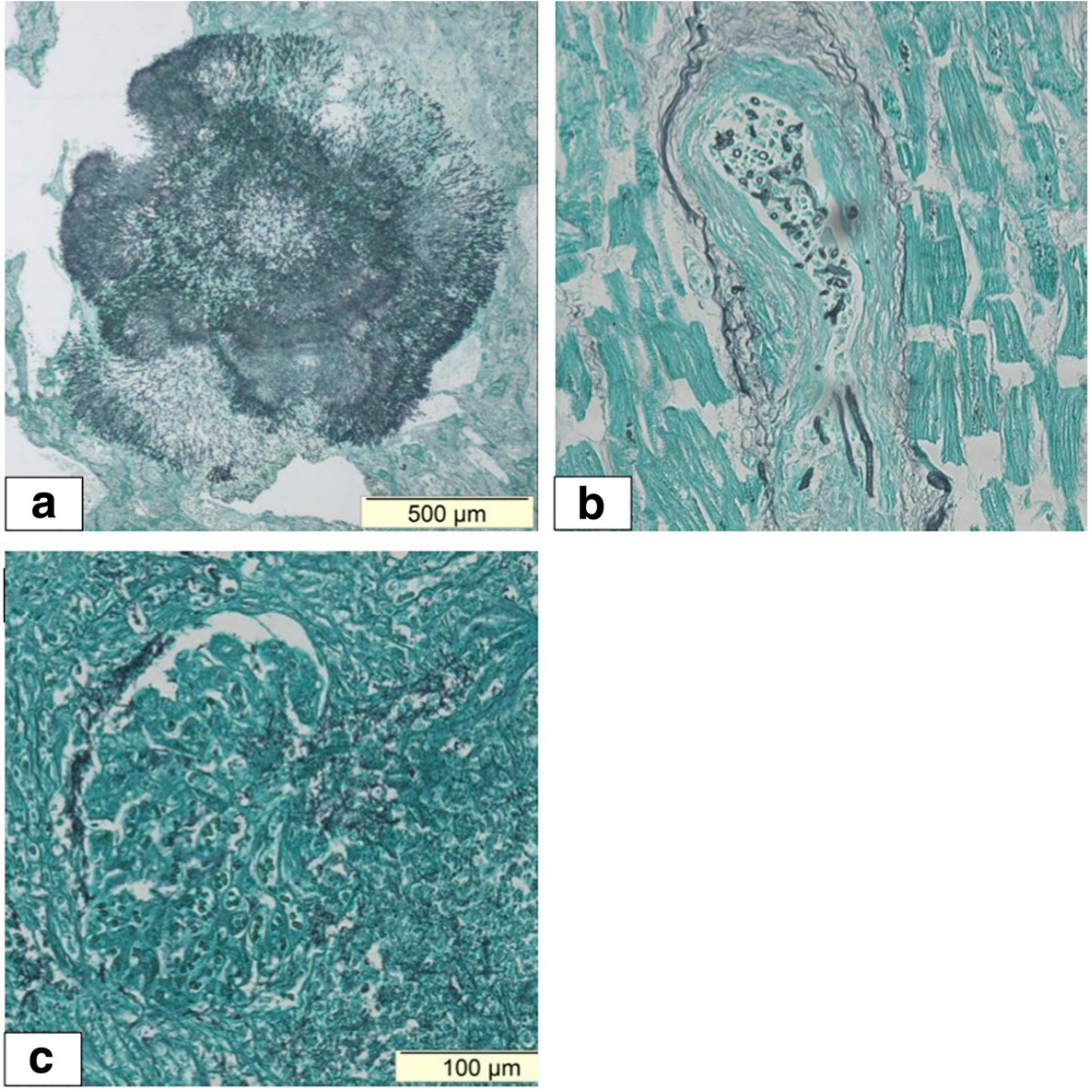
**Autopsy findings. (a)** Mucormycosis in the upper lobe of the left lung. **(b)** Mucormycosis invading a cardiac artery in the septum. **(c)** Nocardia in the glomerulus of the left kidney.

**Figure 3:**
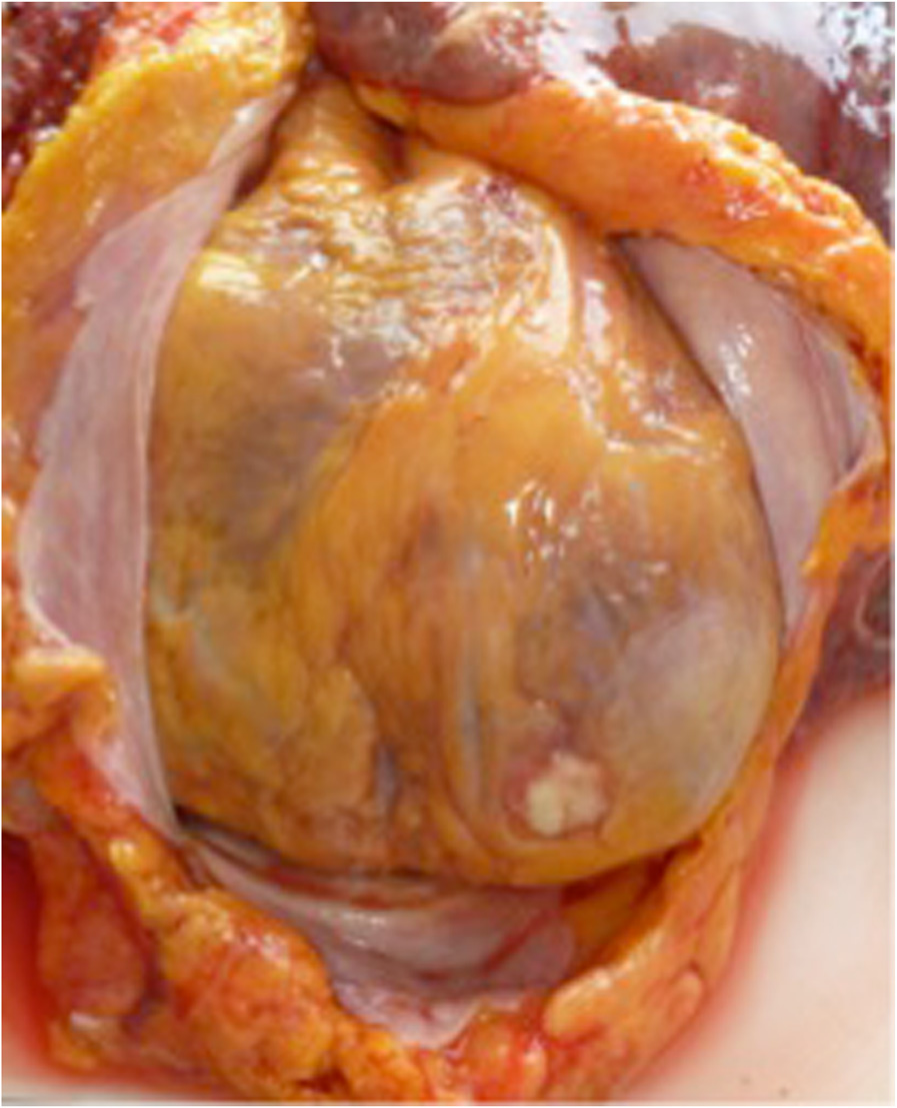
Post-mortem specimen showing abscess formation on the apex of the heart.

Ten days after our patient’s ICU admission she died of multiple organ failure. To uncover the full extent of the infection, an autopsy was performed. A brain autopsy was refused by the family on emotional grounds. Surprisingly, multiple abscesses and necrotic areas containing both *Nocardia* and Mucorales were detected in both lungs (Figure 2a), as well as in her heart (endo-, myo- and pericardium and tricuspid valve; Figure 3) and some septal arteries (Figure 2b). *Nocardia* was also found in both her kidneys (Figure 2c) and her intestines. Behind the left lobe of the thyroid gland an abscess was detected containing *Nocardia*. The *Nocardia* isolate was further identified as *Nocardia farcinica.* Identification was obtained on the basis of sequence determination of the 16S rRNA gene, as described previously [6], and by comparison of the sequence with those obtained in a previous study [7]. Antibiotic sensitivity was tested using the disc diffusion method.

## Discussion

In retrospect, these infections probably accounted for some of the patient’s previous symptoms. This case clearly illustrates the challenges of timely diagnosis of unusual infections. The differential diagnosis was broad and included opportunistic infections, malignancy and interstitial lung disease. The possibility of an opportunistic infection, including mucormycosis, tuberculosis, actinomycosis and nocardiosis, all having similar appearance on CT, ranked high in the list of possible diagnosis [8]. The presence of mildly enlarged hilar and mediastinal lymph nodes, as found in our patient, is unusual in nocardiosis. This finding, together with radiologic signs suggestive of previous tuberculosis exposure and the clinical presentation of night sweats and weight loss further complicated the differential diagnosis. Pulmonary malignancies and interstitial lung disease, including lupus interstitial pneumonia, non-specific interstitial pneumonia or co-existing sarcoidosis, were also part of the diagnostic spectrum.

Multiple factors contributed to the difficulty of the diagnostic process. First, our patient presented with recurrent acute pulmonary infections with temporary improvement after treatment with antibiotics, mostly fluoroquinolones. Fluoroquinolones have activity against *Nocardia farcinica*[2]. Second, it took six months to acquire a positive (blood) culture for *Nocardia.* The microbiological challenge is well described in the literature for nocardiosis and mucormycosis [4,5]. Third, *Pseudomonas aeruginosa* was isolated from the BAL and initially deemed responsible for the infection. Other interfering elements were the signs of heart failure and that the infection mimicked a flare-up of SLE [9]. Importantly, disease activity and infection can occur simultaneously [4].

Isolation of *Nocardia* in any patient, from any body source, should always be regarded as significant [2]. An aggressive diagnostic strategy that includes BAL and tissue biopsies may significantly improve the outcome [4].

Rapid identification of infection with *Nocardia* is crucial, because delayed diagnosis and treatment has an important impact on the prognosis [9]. Because of the evolution to septic shock, ceftriaxone and amikacin were added to our patient’s antibiotic regimen. An optimal antibiotic treatment for nocardiosis is not yet established [2,10]. Trimethoprim-sulfamethoxazole has been the drug of choice for treatment of nocardiosis for many years [5,10,11] but there are several other antibiotics with activity against *Nocardia*, including ceftriaxone, cefotaxime, amikacin, imipenem, meropenem, linezolid, minocycline, moxifloxacin, levofloxacin, tigecycline and amoxicillin-clavulanic acid [2]. Because there is a difference in antibiotic susceptibility pattern between *Nocardia* species, identification to the species level and antibiotic susceptibility testing should, like in our case, be undertaken in each patient and treatment should be individualized accordingly [2]. Combination therapy with a second or even a third antibiotic agent in severe cases can be warranted until the susceptibility pattern is known and the clinical condition of the patient improves [2]. The duration of antibiotic therapy is often prolonged to minimize disease relapse (6 to 12 months, and in some cases even longer) [2,5,10]. The mortality rate in immunosuppressed patients with pulmonary and/or disseminated *Nocardia* infection is as high as 65% [11].

Mucormycosis is a very aggressive invasive fungal disease. When suspected, a biopsy should be obtained whenever possible. Diagnosis requires direct microscopy, histopathology and culturing of clinical specimens [12]. Unfortunately, the pre-mortem diagnostic rate of mucormycosis is extremely low [4]. The mainstay of treatment is surgical debridement and treatment with (liposomal) amphotericin B (at least 5mg/kg/day), or posaconazole (200mg, four times daily) in case of treatment failure with amphotericin B [12].

A handful of cases of mucormycosis in SLE were reported in the English literature and most of them were disseminated infections with fatal outcome even if adequate therapy with amphotericin B was initiated [4]. Only a few cases of combined disseminated mucormycosis and nocardiosis in immunosuppressed patients have been reported [13].

## Conclusions

To the best of our knowledge, there have been no previous cases of combined disseminated nocardiosis and mucormycosis in patients with SLE reported in the literature. Patients with SLE are at high risk for opportunistic infections because of cellular immunosuppression caused by lupus itself or by its treatment. This case is another reminder that we should expect the unexpected (pathogen) in the immunocompromised patient. Both nocardiosis and mucormycosis may mimic other pathologies, on clinical as well as radiological examination. Invasive sampling procedures are often needed to prove infection. Establishing a timely diagnosis is challenging and unfortunately the correct diagnosis is often only made post-mortem.

## Consent

Written informed consent was obtained from the patient's next of kin for publication of this case report and any accompanying images. A copy of the written consent is available for review by the Editor-in-Chief of this journal.

## Abbreviations

BAL: bronchoscopy with bronchoalveolar lavage; HRCT: high-resolution computed tomography; ICU: intensive care unit; SLE: systemic lupus erythematosus.

## Competing interests

The authors declare that they have no competing interests.

## Authors’ contributions

FDC and FVR drafted the manuscript and contributed equally in the writing of this case report. DB, PD, PD and JD suggested final corrections. AH performed the autopsy and provided the images. GC and PC further identified *Nocardia* to the species level and suggested final corrections. All authors read and approved the final manuscript.
